# Magnetic and electronic properties of magnetite across the high pressure anomaly

**DOI:** 10.1038/s41598-019-41184-3

**Published:** 2019-03-14

**Authors:** D. P. Kozlenko, L. S. Dubrovinsky, S. E. Kichanov, E. V. Lukin, V. Cerantola, A. I. Chumakov, B. N. Savenko

**Affiliations:** 1 Frank Laboratory of Neutron Physics, Joint Institute for Nuclear Research, 141980 Dubna, Russia; 2 Bayerisches Geoinstitut, Universität Bayreuth, D-95440 Bayreuth, Germany; 3 European Synchrotron Radiation Facility, BP 220, F-38043 Grenoble, France

## Abstract

The magnetite Fe_3_O_4_, being anciently known magnetic material to human kind and remaining in leading positions for development of advanced technologies presently, demonstrates a number of puzzling physical phenomena, being at focus of extensive research for more than century. Recently the pressure-induced anomalous behavior of physical properties of magnetite in vicinity of the structural phase transition, occurring at *P* ~ 25–30 GPa, has attracted particular attention, and its nature remains unclear. Here we study the magnetic and electronic properties of magnetite across high pressure anomaly and in the pressure-induced phase by means of ^57^Fe synchrotron Moessbauer spectroscopy and neutron diffraction. The hyperfine interaction parameters behavior was systematically analysed over pressure 0–40 GPa and temperature 10–290 K ranges. In the high pressure phase the ferrimagnetic order formation below *T*_NP_ ~ 420 K was observed and spin arrangement symmetry was deduced. The structural, magnetic and electronic phase diagram of magnetite in the discussed pressure range is established.

## Introduction

Magnetite is the most oldest known magnetic mineral, playing important role in natural life and development of technological applications. Starting from the utilization in ancient compasses for navigation about three thousand years ago, now magnetite is used in the development of advanced nanotechnologies for applications in electronic devices, medical diagnostics and treatment, imaging and production of magnetic nanocomposite materials^1–4^.

**Figure 1:**
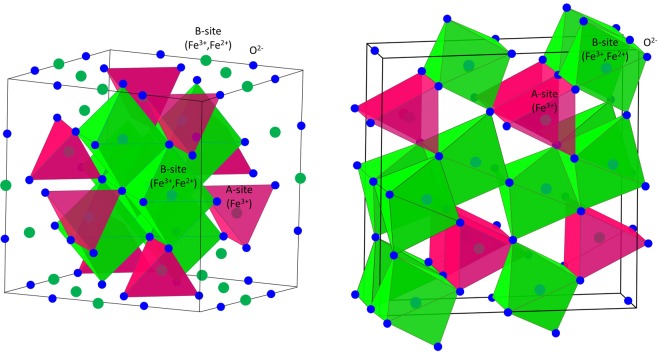
The inverse spinel cubic (left) and post-spinel orthorhombic (right) crystal structure of magnetite. The A iron sites with tetrahedral (in the spinel structure) or prismatic (in the post-spinel structure) oxygen coordination and B iron sites with the octahedral oxygen coordination are shown.

At ambient conditions magnetite crystallizes in the inverse spinel cubic structure (Fig. 1), containing Fe^3+^ ions in the sites with tetrahedral oxygen coordination (A) and mixture of the Fe^3+^ and Fe^2+^ ions in equal proportion in the sites with octahedral oxygen coordination (B). The Fe spins at the A and B sublattices are arranged ferrimagnetically below the Néel temperature, *T*_N_ = 850 K. A presence of the mixed valence iron ions in such a structure leads to challenging physical phenomena arising in magnetite upon temperature and pressure variation, which nature remains a topic of extensive debates over the last few decades, despite the extensive research. Upon temperature lowering, the Verwey transition in Fe_3_O_4_ occurs (*T*_V_ ≈ 120 K), resulting in the abrupt resistivity increase by about two orders of magnitude^5^. The nature of this transition is associated with the complex charge and orbital order, involving trimerons formation and Jahn-Teller active distortions, giving rise to magnetic polarons^6–9^.

An application of high pressure affects anomalously the behavior of magnetic and electronic properties of magnetite, ultimately leading to a structural phase transition above *P* ~ 25 GPa^10–16^. A number of incompatible models were proposed to explain nature of the observed phenomena. The temperature and pressure - mediated charge redistribution over the sites with tetrahedral and octahedral oxygen coordination via the inverse-to-normal transition in the spinel structure, [Fe^3+^]_A_[Fe^2+^Fe^3+^]_B_O_4_ → [Fe^2+^]_A_[Fe^3+^Fe^3+^]_B_O_4_, was proposed, based on the Moessbauer spectroscopy and powder X-ray diffraction data^17,18^. Alternatively, a realization of the high spin (HS) to intermediate spin (IS) transition of Fe^2+^ ions at *P* ~ 12–16 GPa was assumed in the X-ray magnetic circular dichroism (XMCD) and photoemission spectroscopy study^15^. Another mechanism of the enhanced electron delocalization was suggested in the later Moessbauer spectroscopy, single crystal X-ray diffraction and XMCD experiments^16,19^.

While the latest single crystal X-ray diffraction experiments confirmed the orthorhombic CaTi_2_O_4_-type (*Bbmm* symmetry) crystal structure of the high pressure post-spinel phase of magnetite (Fig. 1)^20^, the magnetic and electronic properties of this phase remain unclear. The proposed models range from paramagnetic behavior at ambient temperature to a progressive evolution of non-magnetic component upon compression at low temperature within the magnetically ordered structure^14,21^. In addition, a high spin to low spin (LS) state crossover of Fe^3+^ ions at pressures around 40 GPa was evidenced^20,21^.

In order to explore in detail the magnetic and electronic properties of magnetite across the high pressure anomaly and in the pressure- induced phase, we have performed synchrotron Moessbauer spectroscopy and neutron diffraction measurements over the 0–40 GPa pressure and 10–300 K temperature range. Different response of highly correlated lattice, spin and charge degrees of freedom on variation of thermodynamic parameters enabled to distinguish the characteristic temperatures of the structural and magnetic phase transitions and spin crossover and construct the phase diagram of magnetite in the studied pressure and temperature ranges.

## Results

### Synchrotron Moessbauer source spectroscopy

**Figure 2:**
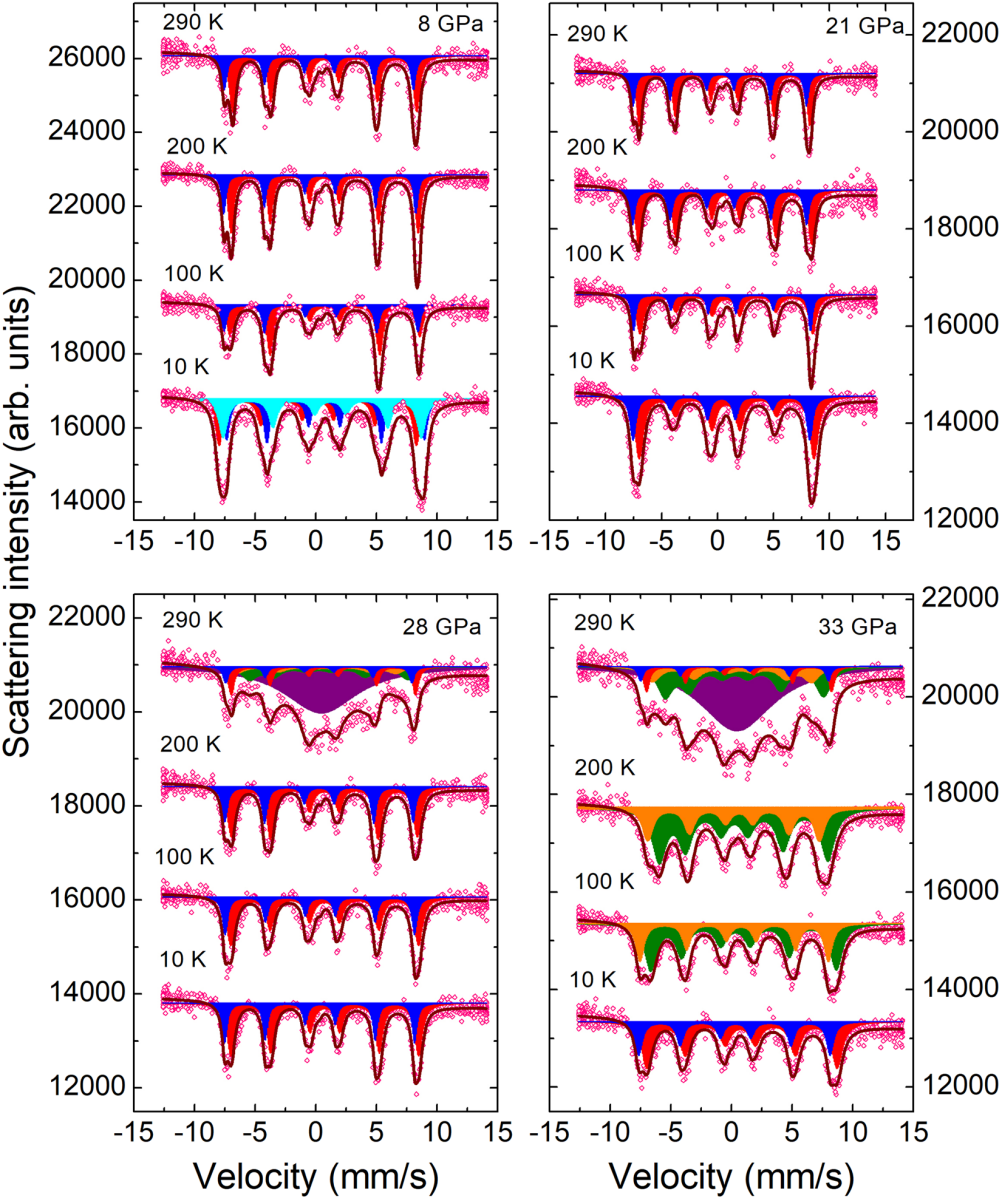
The SMS spectra of Fe_3_O_4_, measured at selected pressures and temperatures. The experimental points, fitting curves and spectral components corresponding to particular iron sites in different structural and magnetic phases are presented. The sextet components, corresponding to the tetrahedral and octahedral sites in the spinel phase, are shown in blue and red colors. At *P* = 8 GPa and *T* = 10 K, additional component associated with the octahedral site, is shown in cyan color. The sextet components, corresponding to the bicapped trigonal prismatic and octahedral sites in the post-spinel phase, are shown in orange and olive colors. The paramagnetic doublet component is shown in purple color.

The synchrotron Moessbauer source (SMS) spectra of single crystalline Fe_3_O_4_ sample (^57^Fe isotope content >90%) measured at selected pressures and temperatures are shown in Fig. 2. The spectra measured at *P* = 8 GPa in the temperature range 290–100 K can be well fitted with the two sextet components, one corresponding to the Fe^3+^ ions in HS state (*S* = 5/2) located in the tetrahedral (A) sites and second one to the mixed Fe^3+^ and Fe^2+^ (*S* = 2) ions in HS state located in the octahedral (B) sites with relative abundance 1:2, respectively. The electronic hopping between the Fe^2+^ and Fe^3+^ ions in the octahedral sites^22^ is characterized by a relaxation time several orders of magnitude less in comparison with the characteristic measurement time of the Moessbauer experiment. This makes them indistinguishable and the relevant iron valence state can be described as an averaged Fe^2.5+^ one. The obtained values of the hyperfine parameters (Supplementary Table 1), isomer shifts *IS*_A_ = 0.28(4) and *IS*_B_ = 0.71(3) mm/s, quadrupole splittings *QS*_A_ = −0.03(7) and *QS*_B_ = 0.04(8) mm/s, and hyperfine magnetic fields *H*_hfA_ = 48.4(2) and *H*_hfB_ = 47.1(2) T at ambient temperature are comparable with those reported in previous studies^19,23^. The larger value of the A-site hyperfine field points to prevalence of the A-sublattice magnetization over the B-sublattice one in the ferrimagnetically ordered state, in accordance with the expected spin-only values of the magnetic moments, 5 and 4.5 µ_B_ respectively^24^.

On cooling down to 100 K, a nearly linear increase of the isomer shift values to *IS*_A_ = 0.41(4) and *IS*_B_ = 0.76(3) mm/s and hyperfine magnetic fields to *H*_hfA_ = 49.6(2) and *H*_hfB_ = 48.4(2) T occur (Supplementary Table 1). Below *T* = 100K, additional splitting of the absorption lines was observed (Fig. 1), associated with the Verwey transition, which leads to complex charge localization phenomena and structural distortions in magnetite. At ambient pressure below the Verwey transition temperature, the observation of five independent sextet components in the Moessbauer spectra, one corresponding to the tetrahedral sites and four to the octahedral Fe^3+^/Fe^2+^ sites with different charge states, was previously reported^25^. Due to restrictions of the high pressure experiments, we were unable to resolve unambiguously all these components and applied a simplified approach involving three sextets, corresponding to Fe^3+^ ions in tetrahedral sites and Fe^3+^ and Fe^2+^ ions in two octahedral sites. The values of isomer shifts *IS*_A-Fe3+_ = 0.29(5), *IS*_B-Fe3+_ = 0.38(3), IS_B-Fe2+_ = 1.03(4) mm/s, quadrupole splittings *QS*_A-Fe3+_ = 0.3(1), *QS*_B-Fe3+_ = −0.6(1) and *QS*_B-Fe2+_ = −0.5(1) mm/s, and hyperfine magnetic fields *H*_hfA-Fe3+_ = 50.6(1), *H*_hfB-Fe3+_ = 50.5(1) and *H*_hfB-Fe2+_ = 50.5(2) T were obtained at *T* = 10 K, which are comparable with the averaged values for the Fe^3+^ and Fe^2+^ ions in relevant groups of sites obtained earlier at ambient pressure^25^.

**Figure 3:**
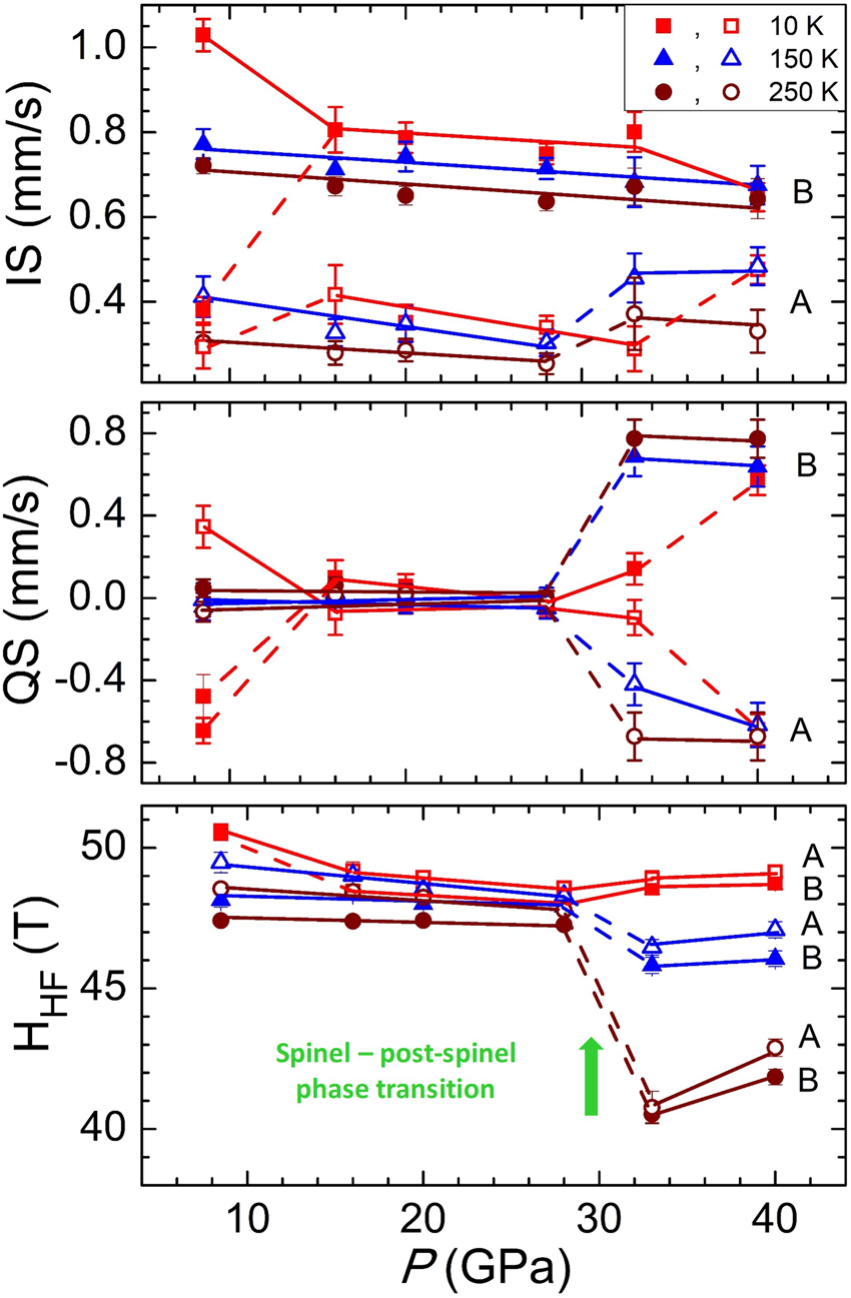
The hyperfine parameters in Fe_3_O_4_ as functions of pressure at selected temperatures 10, 150 and 250 K. The open and solid symbols correspond to (**A**) tetrahedral sites of iron in the spinel phase or bicapped trigonal prismatic sites in the post-spinel phase and (**B**) octahedral sites of iron in both phases.

At pressures above 8 GPa no signatures, characteristic for the Verwey transition, were observed in the SMS spectra (Fig. 2), confirming its suppression^26^. The isomer shifts decrease linearly under pressure with more pronounced absolute changes for the tetrahedral A sites. The quadrupole splittings exhibit weak pressure variation within their determination error. The hyperfine magnetic fields also decrease with similar pressure slopes for both the tetrahedral and octahedral sites (Fig. 3, Supplementary Table 1).

At *P* = 28 GPa and ambient temperature, an appearance of additional broad magnetic sextet components characterizing by the lower hyperfine magnetic field values and paramagnetic doublet is observed (Fig. 2). This observation can be attributed to the onset of the pressure-induced post-spinel phase, which hyperfine parameters are substantially different in comparison to those in the spinel phase, and the spin state crossover.

**Figure 4:**
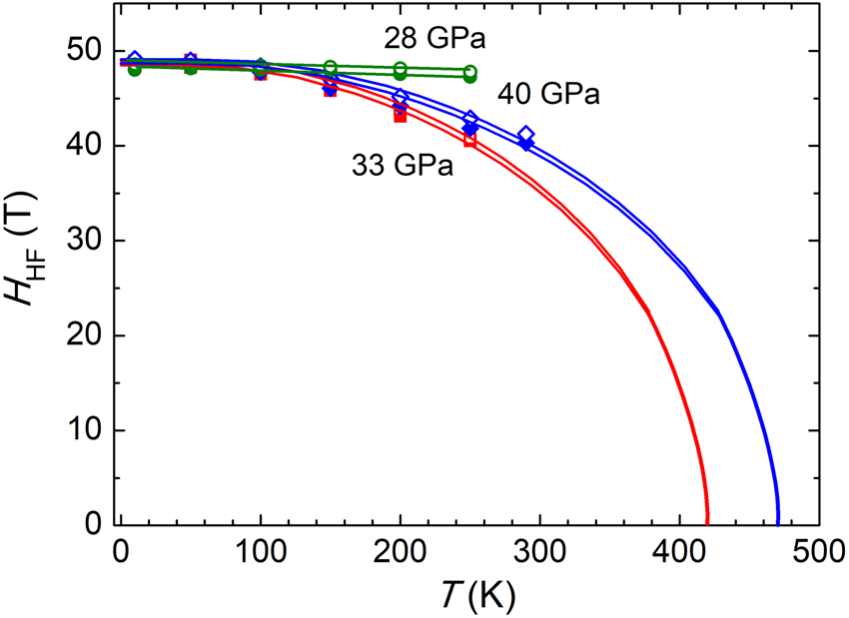
The hyperfine magnetic fields as functions of temperature fitted using the Brillouin functions at *P* = 28, 33 and 40 GPa. The open and solid symbols correspond to Fe^3+^ sites with tetrahedral (28 GPa) or prismatic (33, 40 GPa) oxygen coordination and Fe^2.5+^ sites with the octahedral oxygen coordination, respectively.

Upon compression to 33 GPa at *T* = 290 K the intensity of the new components has grown at the expense of ones corresponding to the spinel phase (Fig. 2). On cooling to *T* = 10 K, the initial spinel phase was restored, as one can conclude from the obtained *IS*, *QS* and *H*_hf_ values, close to those corresponding to lower pressures (Fig. 4, Supplementary Table 1). However, on subsequent warming above 50 K, drastic changes in the SMS spectra occur, hence signaling the phase transformation to the post-spinel high pressure modification (Fig. 2, Supplementary Fig. 1). The spectra obtained in the temperature range 50–250 K, corresponding to the pressure-induced phase, can be successfully described using only two sextet components with relative abundance 1: 2. The hyperfine parameter values *IS*_A_ = 0.49(4) mm/s, Q*S*_A_ = −0.52(7) mm/s and *H*_hfA_ = 49.2(2) for the first component and *IS*_B_ = 0.69(4) mm/s, Q*S*_B_ = 0.57(8) mm/s and *H*_hfB_ = 48.2(2) T for the second component were obtained at *T* = 50 K. No paramagnetic doublet was found at temperatures below 290 K. The observed components can be attributed to the Fe^3+^ ions in the eightfold bicapped trigonal prismatic oxygen coordination (A) and mixed Fe^2.5+^ state in the octahedral oxygen coordination (B) in the orthorhombic post-spinel phase of the *Bbmm* symmetry^13,20^. The modification of the oxygen coordination from tetrahedral to prismatic one at Fe^3+^ sites leads to increased values of the isomer shift, while the relevant value for the octahedrally coordinated Fe^2.5+^ sites remains similar to that in the spinel phase. A more pronounced distortion degree of oxygen polyhedra at both Fe^3+^ and Fe^2.5+^ sites results in noticeably larger quadrupole splitting parameters with respect to those in the cubic spinel phase (Fig. 3). The obtained hyperfine magnetic fields at the iron sites in the post-spinel phase are comparable with those in the initial spinel one, and the value *H*_hfA_ exceeds the *H*_hfB_ one. This implies that the Fe^3+^ and Fe^2+^ ions remain in the HS state in the considered temperature range and the resulting magnetic structure is ferrimagnetic. From the fitting of the temperature dependences of the hyperfine magnetic fields with the Brillouin functions corresponding to spin values *S* = 5/2 for the Fe^3+^ sites and average of *S* = 5/2 and *S* = 2 for the Fe^2.5+^ sites, the magnetic ordering temperature value *T*_NP_ = 420(5) K was obtained for the post-spinel phase, (Fig. 4) which is more than twice smaller with respect to *T*_N_ ≈ 850 K for the spinel phase^27^.

The observation of the additional paramagnetic doublet at 290 K (Fig. 2) can be associated with the temperature induced spin state crossover of Fe^3+^ ions from the high spin HS (*S* = 5/2) to the low spin LS (*S* = 1/2) state in the octahedral sites of the post-spinel phase. It is characterized by parameters *IS* = 0.46(7) mm/s and *QS* ≈ 0.5(1) mm/s at *P* = 33 GPa, compatible with the LS state of Fe^3+^ ions^20,21^.

After cooling the sample to 10 K, we have increased pressure up to 40 GPa. In the temperature range 10–290 K, only sextet components associated to the post-spinel phase were observed in the SMS spectra (Supplementary Fig. 2 and Supplementary Table 1, obtained hyperfine interaction parameters at selected temperatures are also presented in Fig. 3). From the fitting of the temperature dependences of the hyperfine magnetic fields it was found that the magnetic ordering temperature increases up to 475 K with a pressure coefficient *dT*_*NP*_*/dP* ≈ 8 K/GPa. The absence of the doublet component in the data measured at *T* = 290 K implies that onset of the spin state crossover is shifted to higher temperatures.

### Neutron diffraction

**Figure 5:**
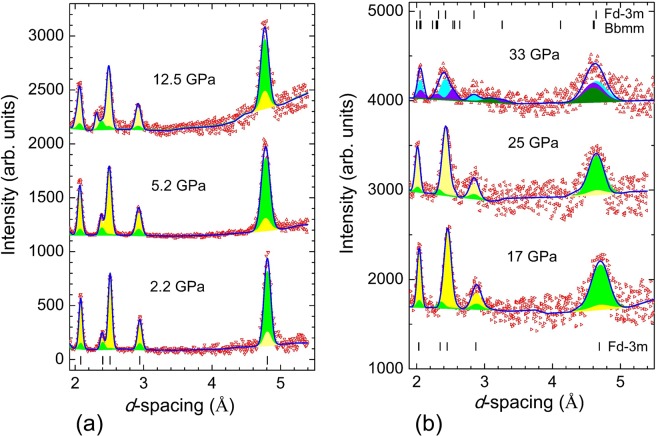
The neutron diffraction patterns of Fe_3_O_4_ measured with the sapphire anvil high-pressure cell up to 12.5 GPa (**a**) and the DAC up to 33 GPa (**b**) at ambient temperature. The experimental points and calculated profiles are shown. Tick marks represent the calculated positions of nuclear peaks for the cubic spinel crystal structure of *Fd* $\bar{3}$ *m* symmetry and the orthorhombic crystal structure of *Bbmm* symmetry (for *P* = 33 GPa). For the diffraction patterns, corresponding to the spinel phase (0–25 GPa), the contributions from nuclear and magnetic structures are shown in yellow and green colors. For the diffraction pattern, measured at *P* = 33 GPa, the total contributions from nuclear and magnetic structures, corresponding to the spinel and post-spinel phases, are shown in cyan and violet colors. For the post-spinel phase, the magnetic contribution is also shown in olive color.

The neutron diffraction patterns of powdered magnetite sample, measured at selected pressures and ambient temperature, are shown in Fig. 5. In the pressure range up to 25 GPa, they correspond to the cubic spinel phase. The long range ferrimagnetic order provides the dominant contribution to the intensity of the peak (111) located at *d* ≈ 4.8 Å, while one from the nuclear scattering to this peak is quite small, as well as partial contribution to the intensity of the peaks (220) at *d* ≈ 2.95, (222) at *d* ≈ 2.40 and (400) at *d* ≈ 2.08 Å (the *d*-spacing values are given for ambient pressure). The obtained values of ordered Fe magnetic moments at ambient pressure, corresponding to the A- and B- magnetic sublattices, *m*_A_ = 4.42(7) and *m*_B_ = 4.33(7) µ_B_, are consistent with results of previous studies^27^.

**Figure 6:**
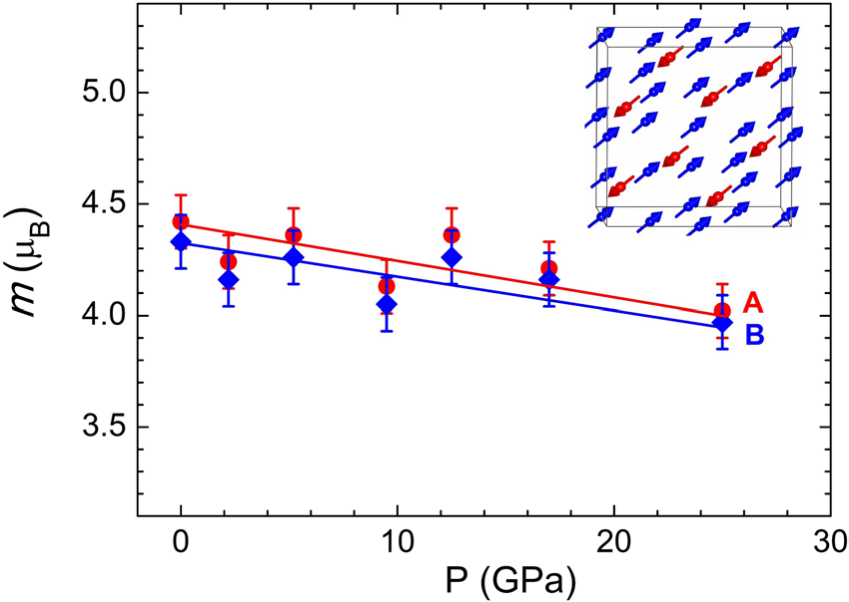
The ordered Fe magnetic moments at the (**A**,**B**)- sublattices as functions of pressure and their linear interpolation. The inset shows the ferrimagnetic structure of the spinel phase.

At high pressures the ordered magnetic moments were evaluated with a restriction of scaling their ratio with those of the relevant hyperfine magnetic fields, known to be proportional each other in magnetite^24^. The obtained ordered Fe magnetic moments are reduced weakly upon compression (Fig. 6), reaching values *m*_A_ = 4.02(8) and *m*_B_ = 3.97(8) µ_B_ at *P* = 25 GPa, respectively.

**Figure 7:**
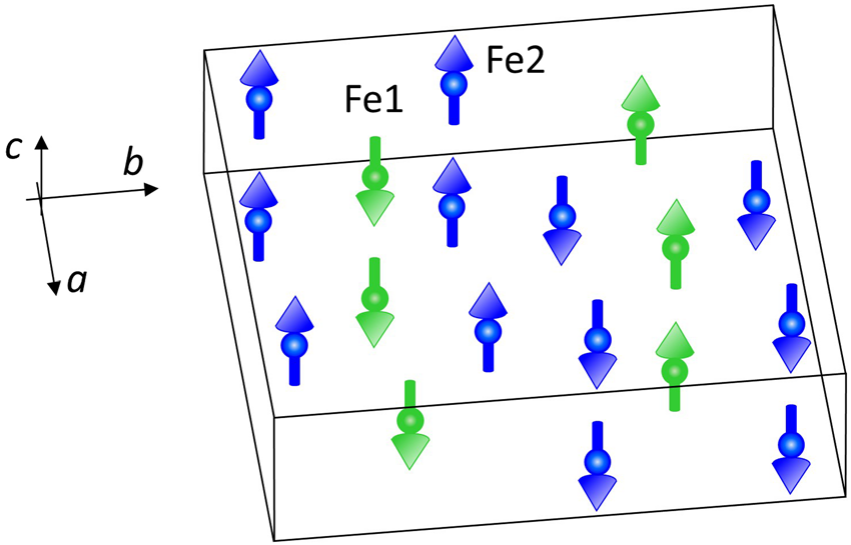
The spin arrangement in the high pressure phase of magnetite. The directions of the magnetic moments of the Fe1 and Fe2 ions are shown.

At *P* = 33 GPa, a redistribution of the diffraction peaks intensity in the *d*-spacing region 2.2–2.4 Å, as well as broadening of the magnetic peak at *d* ≈ 4.62 Å was observed, pointing to the structural phase transition. Such a behavior is consistent with results of the SMS experiments, if compression at ambient temperature is considered. In the Rietveld refinements of the experimental data (Fig. 5), a model involving coexistence of the initial cubic spinel *Fd* $\bar{3}$ *m* phase and the pressure-induced post-spinel orthorhombic *Bbmm* phase was used. The atomic coordinates in the orthorhombic phase (Supplementary Table 2) were fixed to values evaluated from the interpolation of those found in the single crystal and powder X-ray diffraction data at pressures 28 and 41 GPa^13,20^. The refined lattice parameters of the spinel phase, *a* = 8.040(5) Å and post-spinel phase, *a* = 9.246(5), *b* = 9.278(5) and *c* = 2.763(3) Å are consistent with previous results^13,20^. In order to describe magnetic contribution to the experimental data, different possible models of the long range magnetic order in the orthorhombic phase were analysed. Finally, the spin arrangement illustrated in the Fig. 7, was chosen for the fitting of the experimental data. It consists of two antiferromagnetic sublattices formed by the layers of Fe1 and Fe2 ions located in the 4(c) and 8(f) positions with the bicapped trigonal prismatic and octahedral oxygen coordination with the same *y* coordinate and parallel orientation of the magnetic moments, which are stacked antiparallel along the *b*-axis. The overall ferrimagnetic character of this structure is caused by the different magnetic moments of Fe1 and Fe2 ions, as evidenced by the distinctive values of the relevant hyperfine magnetic fields in the SMS experiment. Such a magnetic structure provides the major magnetic contribution to the peak (200)/(020), located at *d* ≈ 4.62 Å and accounts for the observed additional intensity in the given *d*-spacing region. In comparison, the magnetic structure of the initial spinel phase is much simpler and it consists of two ferromagnetic sublattices with antiparallel orientation of the Fe magnetic moments in the A and B sites. The magnetic unit cell coincides with the crystallographic one and the ordered magnetic moments are oriented along the *c*-axis. The effective moment values *m*_*Fe*1_ = 3.00(9) and *m*_*Fe*2_ = 2.98(9) µ_B_ were also evaluated with a restriction of scaling their ratio with those of the relevant hyperfine magnetic fields. For the coexisting cubic spinel phase the effective ordered magnetic moment values *m*_A_ = 3.40(9) and *m*_B_ = 3.35(7) µ_B_ were obtained accordingly. The reduced values of the magnetic moments in the pressure-induced phase are consistent with the spin state crossover detected in the SMS experiments, leading to gradual appearance of the Fe^3+^ (LS) ions with negligible magnetic moments on the B magnetic sublattice with octahedral oxygen coordination.

## Discussion

In the spinel phase, for the temperature (10–290 K) and pressure (8–28 GPa) ranges the *H*_hfA_ value exceeds the *H*_hfB_ one and their relative difference is weakly affected by variation of thermodynamic parameters (Fig. 3, Supplementary Table 1). This rules out suggestions about possible inverse to normal spinel transformation^17,18^ and high spin to intermediate spin state transition of Fe^2+^ ions^15^. The first scenario should lead to reversal of the *H*_hfA_ and *H*_hfB_ values, while the second one should cause the increased difference of these values above the expected HS - IS spin transition pressure point, assumed to occur at *P* ~ 12–16 GPa in^15^ and not observed experimentally in the present study. At the same time, the revealed decreasing trends in pressure behavior of isomer shifts and hyperfine magnetic fields (Fig. 3), as well as ordered magnetic moments (Fig. 6), may be explained by increased hybridization strength between the *d*-orbitals of Fe and *p*-orbitals of O atoms, leading to enhanced electronic delocalization^16,19^. Similar pressure-induced reduction of the ordered magnetic moment was recently also observed in the siderite FeCO_3_ and underlying mechanism was evidenced by the ab-initio calculations^28^.

The *ab initio* calculations have shown that in the post-spinel phase of magnetite the ground state energies corresponding to the ferrimagnetic and antiferromagnetic order have very close values differing by about 0.2 eV only^29^. This implies highly competitive character of magnetic interactions in this structure, leading to the formation of the complex spin arrangement observed experimentally (Fig. 7).

**Figure 8:**
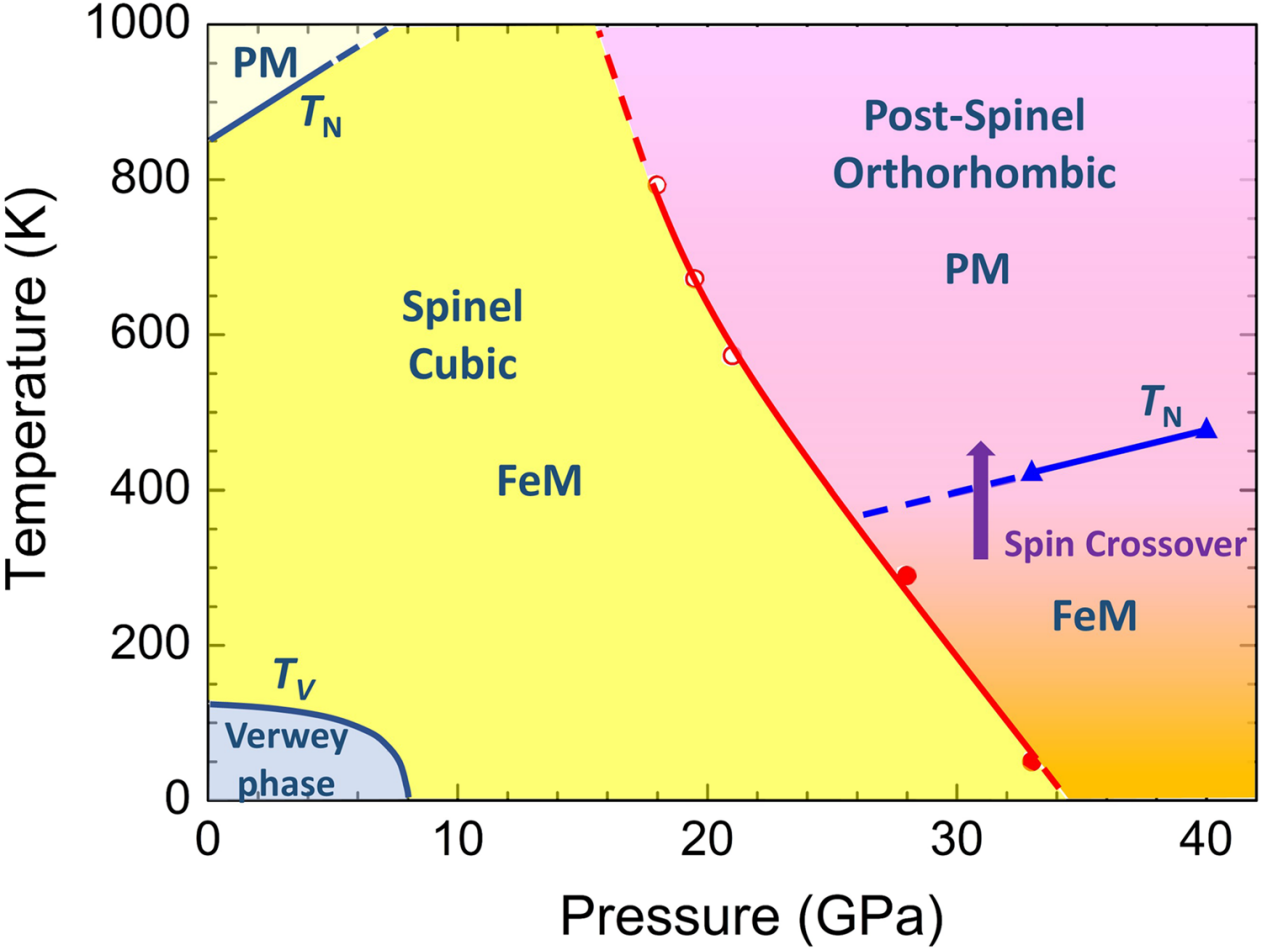
The structural, magnetic and electronic phase diagram of magnetite. The phase boundary of the spinel – post-spinel structural phase transition is constructed using the present results (red solid circles) and data^30^ (red open circles). The pressure dependence of the Néel temperature for the post-spinel phase is obtained from the present data. The pressure dependences of the Néel and Verwey temperatures for the spinel phase are obtained from data^26,31^. The signs “PM” and “FeM” correspond to paramagnetic and ferrimagnetic states.

The structural, magnetic and electronic phase diagram of magnetite, constructed on the base of present data and previous studies^26,30,31^, is shown in Fig. 8. Our evaluations of the spinel – post-spinel structural phase transition points in the studied pressure and temperature ranges are consistent with those obtained at lower pressures and high temperatures^30^. In the spinel phase, the Néel temperature was found to increase with a coefficient *dT*_*N*_*/dP* = 20.5 K/GPa in the pressure range up to 4.5 GPa^31^. The Verwey transition is suppressed at pressures above 8 GPa^26^, in agreement with our observations. In the post-spinel phase, the Néel temperature becomes about twice smaller, and it increases with a noticeably reduced rate *dT*_*NP*_*/dP* ≈ 8 K/GPa. This reduction may be related to a decrease of the leading antiferromagnetic superexchange interaction strength between the A and B sublattices of iron ions via oxygen ions due to a significant reduction of the average value of the Fe(A)-O-Fe(B) bond angle from 123.5° in the spinel phase (*P* = 0 GPa) to about 114.6° in the post-spinel phase (*P* = 33 GPa), as evaluated from the present data (Supplementary Table 2) and results^20^.

The appearance of HS-LS spin state crossover on the magnetic sublattice of Fe^3+^ ions with octahedral oxygen coordination is detected in the vicinity of ambient temperature under pressure (Fig. 8), and it further evolves at elevated temperatures^20,21^.

The present results demonstrate that the long range ferrimagnetic order is formed in the pressure-induced post-spinel phase of magnetite with a more complex spin arrangement and the Néel temperature about twice smaller in comparison with those of the initial spinel phase. The hyperfine interaction parameters in the post-spinel phase are characterized by the substantially increased absolute values of the quadrupole splittings and reduced hyperfine magnetic fields, while variation of the isomer shifts is less pronounced. At temperature *T* ≈ 290 K and pressures above 28 GPa the HS - LS spin state crossover of Fe^3+^ ions evolves on the magnetic sublattice with the octahedral oxygen coordination, leading to the reduction of the effective ordered moments in the magnetic structure.

The different response of highly correlated lattice, spin and charge degrees of freedom to combined variation of thermodynamic parameters (pressure and temperature) allowed to disentangle behavior of the structural and magnetic phase transitions temperatures and the spin crossover, enabling detailed insight into the phase diagram of magnetite.

## Methods

The single crystalline sample of magnetite enriched with the ^57^Fe isotope (>90%) was synthesized as described in^19^. The characterization by the X-ray diffraction and Moessbauer spectroscopy methods confirmed the single phase material without any traces of other iron oxides. The evaluated value of the possible oxygen nonstoichiometry was less than 0.001.

Synchrotron Moessbauer source (SMS) spectroscopy measurements with the single crystalline sample were performed at the Nuclear Resonance beamline^32^ ID18 at the European Synchrotron Radiation Facility (ESRF) using the setup described in^33^. Experiments were carried out from ambient pressure up to 40 GPa, and in the temperature range between 10–290 K. The size of the x-ray beam spot at the sample was about 15 μm in both directions. The BETSA-type membrane diamond anvil cell (DAC) available at ESRF with diamond culets of 250 µm was used. The sample was loaded into the Re gasket indented to about 30 µm thickness with an initial hole of 150 µm diameter. The helium gas was used as a pressure transmitting medium. The pressure was determined by the ruby fluorescence technique using Dewaele calibration scale^34^. The He flow cryostat was used for low temperature measurements. The SMS data were fitted with the MossA software^35^ to obtain hyperfine parameters.

The neutron diffraction experiments were performed with the natural Fe abundance powdered sample of magnetite in the pressure range up to 33 GPa using the DN-6 diffractometer^36^ (IBR-2 pulsed reactor, JINR, Dubna, Russia). The experiments in the pressure range up to 12 GPa were performed using the sapphire anvil high pressure cells and the sample volume of about 2 mm^3^ ^37^. The diffraction patterns were collected at the scattering angle of 90° with the resolution *Δd/d* = 0.015. The experiments in the extended pressure range up to 33 GPa were performed using the diamond anvil cell of Boehler-Almax Plate type. The diamonds with culets of 0.8 mm and aluminium gasket with a hole of 0.4 mm were used. The experimental data were analysed by the Rietveld method using the Fullprof program^38^.

## Supplementary information

Magnetic and electronic properties of magnetite across the high pressure anomaly, supplementary info

## Data Availability

The data that support the findings of this study are available from the corresponding author on request.
