# Supplementary material for: Magnetic and electronic properties of magnetite across the high pressure anomaly
Source: Sci Rep. 2019 Mar 14;9:4464. doi: 10.1038/s41598-019-41184-3 (PMC6418096; doi:10.1038/s41598-019-41184-3)
Supplement: Supplementary file 1 — Magnetic and electronic properties of magnetite across the high pressure anomaly, supplementary info [file 41598_2019_41184_MOESM1_ESM.pdf]

Magnetic and electronic properties of magnetite across the high pressure anomaly

D. P. Kozlenko[1*], L. S. Dubrovinsky[2], S. E. Kichanov[1], E. V. Lukin[1], V. Cerantola[3], A. I. Chumakov[3], and B. N. Savenko[1]

[1] Frank Laboratory of Neutron Physics, Joint Institute for Nuclear Research, 141980 Dubna, Russia

[2] Bayerisches Geoinstitut, Universität Bayreuth, D-95440 Bayreuth, Germany

[3] European Synchrotron Radiation Facility, BP 220, F-38043 Grenoble, France

[*] Corresponding author, e-mail denk@nf.jinr.ru

Supplementary Table 1. The hypefine parameters of magnetite at selected pressures and temperatures.

P, *T*	IS_A (mm/s)	IS_B (mm/s)	QS_A (mm/s)	QS_B (mm/s)	H_{hfA} (T)	H_{hfB} (T)
8 GPa						
290 K	0.28(4)	0.71(3)	-0.03(7)	0.04(8)	48.4(2)	47.1(2)
250 K	0.30(2)	0.72(2)	-0.07(4)	0.05(3)	48.5(2)	47.4(1)
200 K	0.34(2)	0.72(2)	-0.01(2)	0.03(2)	49.1(2)	47.8(1)
150 K	0.41(4)	0.77(3)	-0.01(6)	-0.04(5)	49.5(4)	48.1(2)
100 K	0.41(4)	0.76(3)	0.004(5)	-0.02(4)	49.6(2)	48.4(2)
50 K	0.38(3)	0.82(5)	-0.03(7)	-0.04(7)	50.4(2)	50.3(2)
10 K	0.29(5)	0.38(3) (Fe^{3+})<br>1.03(4) (Fe^{2+})	0.3(1)	-0.6(1) (Fe^{3+})<br>-0.5(1) (Fe^{2+})	50.6(1)	50.5(1) (Fe^{3+})<br>50.5(2) (Fe^{2+})
21 GPa						
290 K	0.22(3)	0.63(2)	-0.04(5)	-0.01(4)	48.0(2)	47.2(2)
250 K	0.29(3)	0.65(2)	0.02(4)	0.01(4)	48.2(2)	47.4(2)
200 K	0.22(4)	0.70(3)	-0.08(8)	-0.04(6)	48.2(3)	47.9(2)
150 K	0.35(4)	0.74(3)	-0.01(6)	0.01(6)	48.5(2)	48.0(2)
100 K	0.41(3)	0.77(4)	0.00(5)	0.04(7)	49.0(2)	47.8(2)
50 K	0.43(3)	0.78(4)	-0.10(6)	0.10(7)	48.9(2)	47.8(2)
10 K	0.35(4)	0.79(4)	-0.01(7)	0.06(6)	48.9(2)	48.4(2)
33 GPa						
290 K<br>spinel<br>post-spinel	 0.26(8)<br>0.34(7)	 0.60(8)<br>0.73(7)	 -0.08(9)<br>-0.32(10)	 -0.12(9)<br>0.66(10)	 47.8(6)<br>40.2(8)	 47.3(6)<br>38.5(8)
250 K	0.37(5)	0.68(5)	-0.52(10)	0.89(10)	40.8(3)	40.5(3)
200 K	0.40(5)	0.62(5)	-0.43(10)	0.78(10)	43.8(3)	43.1(3)
150 K	0.44(5)	0.67(5)	-0.47(9)	0.63(9)	46.5(3)	45.9(3)
100 K	0.50(5)	0.65(5)	-0.60(9)	0.63(9)	48.2(2)	47.5(2)
50 K	0.49(5)	0.69(5)	-0.52(7)	0.57(8)	49.2(2)	48.2(2)
10 K	0.29(5)	0.80(5)	-0.09(7)	0.14(7)	48.9(2)	48.6(2)
40 GPa						
290 K	0.39(5)	0.64(5)	-0.55(10)	0.77(1)	41.2(3)	40.3(3)
250 K	0.33(5)	0.64(5)	-0.66(10)	0.77(10)	42.9(3)	41.9(3)
200 K	0.42(4)	0.65(4)	-0.67(9)	0.71(9)	45.2(3)	44.2(3)
150 K	0.48(4)	0.67(4)	-0.62(9)	0.64(9)	47.1(3)	46.1(3)
100 K	0.46(3)	0.66(3)	-0.62(7)	0.59(7)	48.4(2)	47.8(2)
50 K	0.46(3)	0.67(3)	-0.55(7)	0.56(7)	49.0(2)	48.4(2)
10 K	0.48(3)	0.65(3)	-0.64(7)	0.57(7)	49.1(2)	48.7(2)

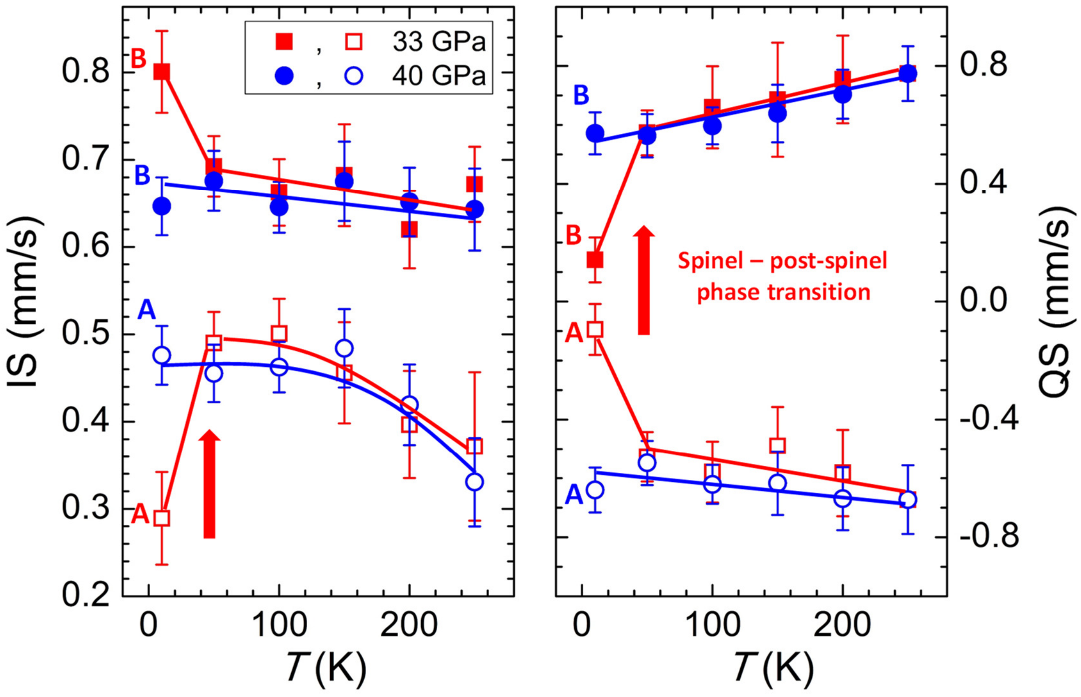

Supplementary Figure 1. Temperature behavior of the isomer shifts and quadrupole splittings of Fe_3O_4 at P = 33 and 40 GPa. The open and solid symbols correspond to (A) tetrahedral sites of iron in the spinel phase or bicapped trigonal prismatic sites in the post-spinel phase and (B) octahedral sites of iron in both phases. The spinel – post-spinel phase transition point is marked by arrow.

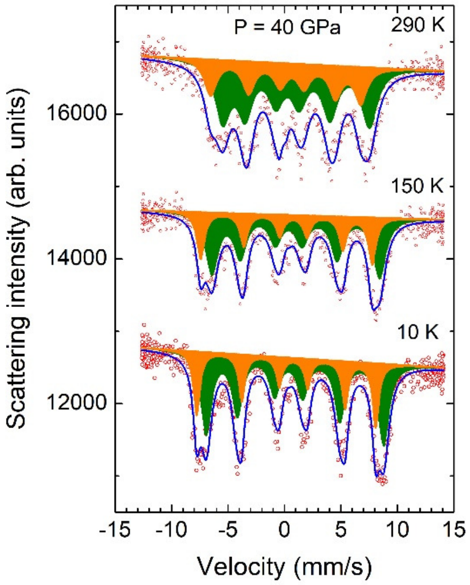

Supplementary Figure 2. The SMS spectra of Fe_3O_4, measured at P = 40 GPa and selected temperatures. The experimental points, fitting curves and the sextet components, corresponding to the bicapped trigonal prismatic (orange color) and octahedral sites (olive color) in the post-spinel phase are presented.

Supplementary Table 2

Structural parameters of the post-spinel phase of Fe_3O_4 with the orthorhombic crystal structure of *Bbmm* symmetry at P = 33 GPa and ambient temperature.

		a (Å)	*b* (Å)	*c* (Å)
Lattice parameters		9.246(5)	9.278(5)	2.763(3)
		Coordinates		
Atom type	Position	*x*	*y*	*z*
Fe1	4*c*	0.377	0.25	0
Fe2	8*f*	0.131	0.077	0
O1	4*b*	0.5	0	0
O2	4*c*	0.033	0.25	0
O3	8*f*	0.215	0.616	0
